# Not so great expectations: The role of price and name information in the nocebo effect

**DOI:** 10.1016/j.rcsop.2025.100630

**Published:** 2025-06-28

**Authors:** Kiarne Humphreys, Michelle Lin, Kirsten Barnes, Yasmin Hasan, Ashwin Vignaraja, Kritika Sarna, Andrew L. Geers, Kate Faasse

**Affiliations:** a School of Psychology, University of New South Wales, Sydney, NSW, Australia; b Department of Psychology, University of Toledo, Toledo, OH, USA

**Keywords:** Nocebo effect, Expectations, Generic medication, Side effects, Price, Drug names

## Abstract

**Background:**

The perception of taking a generic medication can result in reduced efficacy and increased side effects, despite equivalence to brand name medications under double blind conditions. It may be that cues typically associated with generics, including lower price and more complex name, exacerbate negative expectations and cause nocebo effects.

**Methods:**

Healthy participants (*N* = 196) were randomised to receive sham-oxytocin nasal spray associated with either a generic (complex name, low price; *n* = 66) or brand (simple name, high price; *n* = 68) cue, or to no treatment control (*n* = 62). Participants were informed that oxytocin could enhance trust and cooperation, but might cause side effects of headache, nausea, vomiting, rapid heartbeat, feeling faint or light-headed, and skin itching or rash. Treatment-related side effects were assessed at baseline, and after 3 days of sham-oxytocin use.

**Results:**

Nocebo effects were observed across sham-treated participants relative to control (*p* = .015; *d* = 0.28). Association with a generic relative to branded cue significantly enhanced nocebo effects (*p* = .042; *d* = 0.36). Negative expectations mediated the observed nocebo and branding effects.

**Conclusions:**

Cues associated with generic medications can exacerbate nocebo effects and these findings may explain clinical observations of increased side effects from generic medications. Results have important implications for medical care, and interventions to mitigate nocebo effects from generic medications are needed.

Medication use is crucial to preventing, treating, and curing various diseases and conditions. Generic medications are pharmacologically identical versions of branded medications, produced once the original brand patent has expired. Generics provide consumers with a safe and effective treatment option at an affordable price,^1^ and are therefore a necessary part of the healthcare industry.^2^ Evidence from meta-analysis and systematic review support their use. Under double-blind conditions (i.e., when individuals are unaware of the type of medication they are using), generic medications typically demonstrate clinical equivalence to their branded counterparts in terms of quality, safety, and efficacy.^3^ However, in clinical settings and experimental studies lacking double-blind conditions (i.e., when individuals know the type of medication they are using), complications can arise.^4^^,^^5^

The perception that a medication is generic can result in poorer treatment outcomes including increased side effect reporting.^4^^,^^6^^,^^7^ Clinical research has shown that patients who used a pain relief medication with generic labelling (i.e., more complex name, lower price, plain packaging) were more likely to discontinue the medication than patients who used the same medication with brand labelling (i.e., simpler brand name, higher price, known manufacturer, original packaging^7^). Strikingly, experimental research demonstrates that participants report more side effects from a placebo treatment labelled as generic rather than branded.^4^^,^^8^ Given that these treatments were placebos (i.e., pharmacologically inert), the medication itself could not cause these side effects. Instead, increased side effects were likely the result of a psychobiological phenomenon known as the nocebo effect.

The nocebo effect refers to the experience of adverse outcomes - including unpleasant and harmful side effects - caused by the treatment context rather than by the pharmacological properties of the treatment itself.^9^, 10., 11, 12 It is theorised that negative expectations about side effects explain the formation of nocebo effects.^10^^,^^13^^,^^14^ Negative expectations about side effects amplify an individual's search for unpleasant symptoms which can result in the increased experience, recognition, and reporting of side effects.^6^^,^^9^^,^^15^^,^^16^ Given that approximately 20 to 35 % of the general population hold negative beliefs about generic medications (e.g., they are less safe, lower quality, and more likely to cause side effects than branded medications), the contextual cues that indicate a medication is generic may trigger negative expectations about treatment outcomes and lead to the increased reporting of side effects.^17^, ^18^, ^19^ Despite negative beliefs about generics being commonplace, there has been no systematic exploration of whether increased side effects associated with generic medicines might be caused by a nocebo effect.^1^^,^^5^

The current literature posits that cues within the treatment context can trigger the negative expectations that contribute to the nocebo effect.^14^^,^^20^ Cues associated with generic medications may trigger such negative expectations. Medication name and price are two of the first cues that patients encounter that indicate whether a medication is generic or branded. While branded medications have a short, simple proprietary name, generic medications are named using their active pharmaceutical ingredient.^21^ Accordingly, generic names are more complex: they are longer, contain many syllables, are difficult to pronounce, and are often difficult to remember.^22^^,^^23^ Furthermore, generic medications are significantly cheaper than branded counterparts, by an estimated 20–90 %.^21^ Some evidence suggests that both these contextual cues – complex name and low price – are associated with heightened negative expectations.24., 25, 26 A complex medication and low medication price may contribute to the formation of nocebo side effects in generic medications due to these negative expectations. Thus, the current study aimed to investigate whether generic-like name and price cues (versus brand-like cues) trigger negative expectations and subsequent nocebo effects.

A novel treatment paradigm was used to induce nocebo effects in this study. Participants were recruited for a four-day study purportedly examining the influence of two oxytocin nasal spray medications on feelings of trust and cooperation. In reality, sham nasal sprays containing only saline were used. Participants were warned that these sprays might cause several side effects: headache, nausea, vomiting, rapid heartbeat, feeling faint or light-headed, and skin itching or rash. Participants were randomised to one of three conditions (no treatment control, brand cue, generic cue), and one of two information types (name information, price information). The no treatment control condition is necessary to assess the presence and magnitude of the nocebo effect.^11^ Negative side effect expectations were measured prior to treatment administration, and warned side effect symptoms were measured at baseline and after 3 days of treatment to assess the nocebo effect.

We hypothesised that: 1) sham treatment administration would result in an overall nocebo effect; 2) generic-like cues (i.e., complex name or low price) would result in greater warned side effect symptoms scores than brand-like cues (i.e., simple name or high price); 3) allocation to a sham treatment condition would result in greater negative expectations; 4) generic-like cues would result in greater negative expectations than brand-like cues; 5) negative expectations would mediate the nocebo effect; 6) side effect expectations would mediate the difference in warned side effect symptoms reported in response to generic- and brand-like cues.

## 1 Methods

### 1.1 Design

**Fig. 1:**
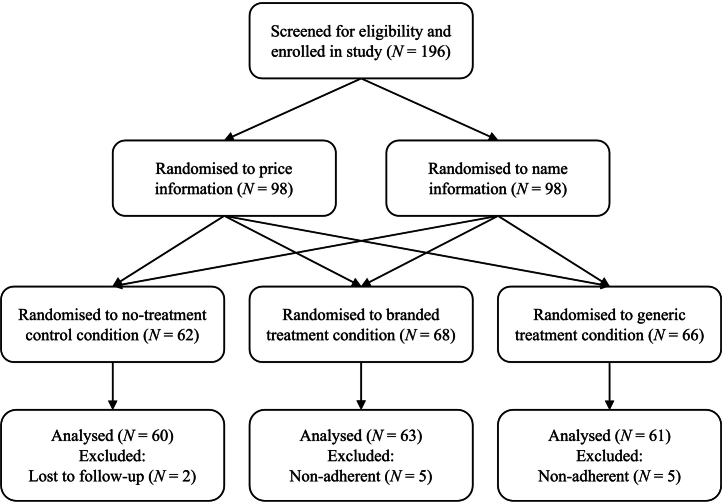
Flow diagram showing the progression of participants through the study.

The study used a 3 (condition: no treatment control, brand cue, generic cue) x 2 (information: name, price) between-subjects experimental design, and consisted of a 30-min face-to-face baseline session, a three-day take-home treatment schedule, and a 10-min online follow-up questionnaire. Participants were first randomised to receive information that described the difference between the two sham nasal sprays either in terms of name (*n* = 98, brand name: Halpam, generic name: Halyazeiiopam) or price (*n* = 98, brand price: $30.33, generic price: $2.43). Within information, participants were randomly allocated to one of three sham treatment conditions: no-treatment control (*n* = 62), branded treatment (*n* = 68), and generic treatment (*n* = 66; see Fig. 1). Block randomisation (blocks of 12) was used to allocate participants to condition and created conditions of equal size. Experimenters strictly followed a script to ensure consistency of information provision across participants. Ethics was granted by the [redacted for peer review]. Data were collected between 31 May and 12 December 2022.

### 1.2 Participants

Participants (*N* = 196) enrolled in a study that ostensibly investigated the effect of oxytocin nasal spray on trust and cooperation. In reality, sham treatments containing only saline solution were employed. Participants were recruited from an undergraduate participant pool (*n* = 68) and the general population (*n* = 128). Based on the results of Faasse and colleagues,^8^ we powered the study to detect a medium effect (*d* = 0.53) in warned side effect symptoms between branded and generic treatments, with power of 0.80 and an alpha of 0.05. A minimum of 30 participants per group (*N* = 180) were required. Participants were ineligible if they were under 18 years of age, not fluent in English, or sensitive or allergic to saline. Additional exclusion criteria were in accordance with the cover story: currently using oxytocin medication, sensitive or allergic to oxytocin, using medications contraindicated with oxytocin (e.g., Zofran, Ephedrine, or Mefloquine), or pregnant or breastfeeding. Of those enrolled, two participants did not complete the follow-up questionnaire, and ten were non-adherent i.e., did not use the nasal spray daily for three days (two participants missed all three days, two missed two days, and six missed one day). The final participant sample comprised 184 participants (see Fig. 1).

The mean age of participants in the final sample was 22.6 years (*SD* = 7.0; range = 18–76). In total, 105 participants identified as female (57 %), 71 as male (39 %), and eight as another gender or preferred not to say (4 %). Approximately half (51 %) of participants were born in Australia, and two-thirds (66 %) spoke English as their first language. Most participants (64 %) reported their highest completed level of education as high school, 31 % as tertiary, and 1 participant chose not to answer. For recompense, participants recruited from the undergraduate pool received partial course credit, and participants from the general population received an AUD$25 gift voucher. Participants were debriefed about the true nature of the study after data collection.

### 1.3 Materials and measures

#### 1.3.1 Side effect information provision

All participants were warned that the nasal sprays could.

cause six possible side effects: headache, nausea, vomiting, rapid heartbeat, feeling faint or light-headed, and skin itching or rash. These symptoms were chosen as they corroborate the cover story (i.e., these side effects are specific to oxytocin use), and they are commonly experienced as medication side effects.^27^^,^^28^ Side effect warnings were provided in written form (i.e., on the Participant Information Sheet) and verbally by researchers.

#### 1.3.2 Sham Nasal spray treatment

Nasal sprays consisted of a bottle that contained approximately 5 mL of medical-grade saline. Condition-specific information was presented on a white label, prominently displayed on the front of the bottle. Participants in the generic condition received either generic name (“Halyazeiiopam”) information, or generic price ($2.43) information on the label. Participants in the branded condition received either branded name (“Halpam”) information, or branded price ($30.33) information on the label. Names were selected from those used by Cho.^24^ Selection of both names and prices was based on ratings in a pilot study (see Appendix 1). Each nasal spray label also identified the spray as containing “Oxytocin”, displayed a fictitious batch number, expiry date, and cautionary advisory statement (e.g., intranasal use only, keep away from children). Those randomised to the no-treatment control condition did not receive a nasal spray.

### 1.4 Primary and secondary outcomes

Primary and secondary outcomes were measured during the in-person session and again at follow-up.

#### 1.4.1 Physical symptoms

A modified version of the Generic Assessment of Side Effects (GASE) scale was employed to measure the subjective experience of symptoms.^27^^,^^29^ Participants rated their experience of 12 symptoms over the past three days on a scale from 0 (*not at all*) to 10 (*very much*). Six symptoms were those that participants were warned about as treatment side effects: headache, nausea, vomiting, rapid heartbeat, feeling faint or light-headed, and skin itching or rash. The remaining six symptoms were used as unwarned control symptoms that were never mentioned, and were matched in population prevalence to the warned side effect symptoms.^30^ These were: feeling tired or fatigued, muscle twitching, hair loss, feeling hot or flushed, muscle weakness, and dry mouth. Warned and unwarned symptoms scores were summed separately.

#### 1.4.2 Expectations

A single-item measure of side effect expectations was modified from the Treatment Expectation Questionnaire (TEX-Q^31^^,^^32^). Wording was as follows: “to what extent do you expect to experience side effects from using/not using the oxytocin nasal spray”. Participants responded on a scale from 0 (*no expected side effects*) to 10 (*high expected side effects*). This item was presented with 10 other questions ostensibly assessing the quality and understanding of treatment information provision to obscure the study's true aims. Expectations were assessed after treatment information provision and condition allocation during the baseline session.

### 1.5 Manipulation and compliance checks

#### 1.5.1 Treatment condition

At follow-up, participants were first asked, “did you receive a nasal spray as a part of this study?” (*yes* or *no*)*.* All participants reported their treatment condition correctly.

#### 1.5.2 Branded vs generic information

To assess the success of the branded vs generic information manipulations, participants randomised to the sham treatment conditions who received price information were asked “how cheap or expensive do you think the medication was?” on a 7-point scale from 1 (*very cheap*) to 7 (*very expensive*). Those randomised to the sham treatment conditions who received name information were asked “how simple or complex do you think the medication name was?” on a 7-point scale from 1 (*very simple*) to 7 (*very complex*). Finally, sham treated participants were asked to select whether they thought the nasal spray was “generic”, “branded”, or if they were “unsure”.

#### 1.5.3 Cover story check

At follow-up, participants responded in a text box to the question “what do you think this study was investigating?” to assess the success of the cover story. Participants whose answers mentioned placebo or nocebo effects, or the impact of name or price information were deemed suspicious of the cover story. In total 8.7 % of participants (*n* = 17) were deemed suspicious. Only one participant in the control condition was deemed suspicious, compared to seven participants in the branded condition, and nine in the generic condition. All primary analyses were run with and without these suspicious participants, and the pattern of results remained unchanged. To maximise power, the reported results include all participants.

#### 1.5.4 Adherence

At follow-up, participants in the sham treatment conditions were asked “how many days did you administer the nasal spray as directed?” on a multiple-choice question (“0 days,” “1 day,” “2 days,” “3 days”). Participants were excluded if they did not use the inert spray for three days.

### 1.6 Demographics and filler items

#### 1.6.1 Demographics

Demographic information included the participants' age, gender, ethnicity, birthplace, first language spoken, and highest education level.

#### 1.6.2 Filler items

At baseline, participants answered questions from the Social Connectedness Scale, General Trust Scale, and Personal and Social Responsibility scale on separate Likert-type scales.^33^, ^34^, ^35^ At follow-up, participants completed adapted versions of the Trust Investment Task, Trustworthy Face Rating Task, Public Project Game, and Dictator game.^36^, ^37^, ^38^ These filler items were included to increase the believability of the cover story and were not analysed.

### 1.7 Procedure

At the beginning of the face-to-face session, participants were informed that the study investigated the impact of intranasal oxytocin on trust and cooperation. The primary researcher assessed the participant's eligibility; those eligible provided electronic informed consent. Participants then completed measures concerning demographics, trust and cooperation, and baseline symptoms. Upon completion, the primary researcher vacated the laboratory to ensure that blinding was maintained.

The secondary researcher entered and informed participants that two nasal sprays were being investigated. Half of the participants were told that the two nasal sprays were named Halpam and Halyazeoiipam (name information). The other half were told that the two nasal sprays cost around $2 and $30 (price information). All participants were informed that both nasal sprays contained the same active ingredient – oxytocin – at the same dosage and were informed of the six potential side effects. The secondary researcher then opened an opaque envelope containing the condition allocation. In the sham treatment conditions, participants received a nasal spray labelled with the appropriate name (Halpam or Halyazeoiipam) or price ($2.43 or $30.33). They received a demonstration regarding how to administer the nasal spray, and were asked to self-administer two sprays per nostril every morning between 6 and 11 am for the next three days. Participants in the no-treatment control conditions did not receive a nasal spray and were informed of their role as control participants (i.e., that their data would act as a control to assess the effects of the nasal spray). All participants were informed they would be emailed a follow-up questionnaire in three days (i.e., the third day of the treatment schedule).

The secondary researcher then left the room and the primary researcher re-entered to complete the baseline session. Participants completed the final evaluation questionnaire, which contained the negative expectation item, and were thanked for their time and reminded about completing the online follow-up questionnaire in three days. Participants received condition-specific text message reminders each day leading up to the follow-up questionnaire. On day three at 5 pm, participants received an email link to the follow-up questionnaire, which included three measures: cover story trust and cooperation tasks, self-report symptom experience over the previous three days, and manipulation and compliance check questions. All participants completed the follow-up questionnaire within 48 h.

### 1.8 Statistical analyses

ANOVA and Chi square tests were used to test for any demographic differences between groups at.

baseline. Given a non-Gaussian right-skewed distribution with an over-representation of zero scores of symptom score and expectations outcomes, Generalised Linear Models (GLM) with a Tweedie distribution and a log link function were employed.^39^ The Tweedie variance power parameter for each model was ascertained by varying this parameter in increments of 0.1 and examining Goodness of Fit statistics (baseline warned symptoms: 1.4, follow-up warned and unwarned symptoms: 1.3, negative expectations: 1.2).

To assess primary outcomes, overall Tweedie models covarying for mean-centred baseline scores were run that contained the main effects of condition (no-treatment control, generic treatment, brand treatment) and information (price vs name) as well as the condition by information interaction on warned symptoms scores. Orthogonal contrasts at the level of the condition factor were used. The first contrast (the nocebo contrast) concerned an overall nocebo effect (i.e., the no-treatment control vs. the two sham-treated conditions; H1). The second (the brand-generic contrast) concerned the difference between treatments (i.e., generic treatment vs. the brand treatment; H2). This same analysis approach was used to assess unwarned symptoms scores, as well as side effect expectations, using the same planned contrasts as above to test H3 and H4.

Based on significant differences in the warned symptoms scores and expectations primary outcomes, mediation analyses were run using the PROCESS macro^40^ with 5000 bootstrap samples and planned orthogonal contrasts as above to test whether the nocebo effect was mediated by negative expectations (nocebo contrast; H5), and whether the difference between the warned symptoms scores of the branded and generic treatment conditions was mediated by mean centred negative expectations (brand-generic contrast; H6), controlling for mean-centred baseline warned symptoms.

Independent samples *t*-tests were conducted to assess differences in perceived name complexity (i.e. was the complex name perceived significantly more complex than the simple name?) and perceived price (i.e. was the low price perceived as significantly lower than the high price?). A chi-square analysis assessed group differences in participants' perceptions of whether medications were generic or branded.

SPSS version 26 was used to conduct all analyses, and statistical significance was set at *p* < .05.

## 2 Results

### 2.1 Descriptive statistics

#### 2.1.1 Baseline demographics

Age did not differ significantly by condition or information, *p*s > 0.10. Similarly, participant gender, place of birth, English as first language, and highest level of education did not differ by condition within information manipulation, *p*s > 0.14 (see Table 1).

**Table 1:** Demographics by group.

	Name Information			Price Information		
	*Control* *(n = 30)*	*Brand* *(n = 32)*	*Generic* *(n = 30)*	*Control* *(n = 30)*	*Brand* *(n = 31)*	*Generic* *(n = 31)*
Age (*M*, *SE*)	21.8 (1.3)	25.8 (1.2)	22.0 (1.3)	22.1 (1.3)	22.4 (1.2)	21.4 (1.2)
Gender (*n, %*)						
Male	10 (33 %)	20 (63 %)	11 (37 %)	7 (23 %)	13 (42 %)	10 (32 %)
Female	18 (60 %)	11 (34 %)	18 (60 %)	22 (73 %)	15 (48 %)	21 (68 %)
Other/not stated	2 (7 %)	1 (3 %)	1 3 %)	1 (3 %)	3 (10 %)	0 (0 %)
Born in Australia (*n*)	17 (57 %)	15 (47 %)	16 (53 %)	16 (53 %)	14 (45 %)	16 (52 %)
English first language (*n*)	20 (67 %)	21 (66 %)	20 (67 %)	17 (57 %)	19 (61 %)	24 (77 %)
Highest education (*n*)						
High school	23 (77 %)	20 (63 %)	22 (73 %)	18 (62 %)	22 (71 %)	21 (68 %)
Tertiary	7 (23 %)	12 (38 %)	8 (27 %)	11 (38 %)	9 (29 %)	10 (32 %)

#### 2.1.2 Baseline symptoms

At baseline, there was not a significant difference in warned symptoms scores by condition or information manipulation (*p*s > 0.54). The information by condition interaction obtained threshold significance, Wald *χ*^2^ = 6.00, *df* = 2, *p* = .050, reflecting differences between the price information control condition and both the name information control condition and the price information brand condition (*p*s < 0.048). Similarly, there was not a significant difference in unwarned symptoms scores by condition or information manipulation (*p*s > 0.25). There was an interaction between condition and information, Wald *χ*^2^ = 6.07, *df* = 2, *p* = .048, reflecting differences between the price information brand condition and each of the price information control condition, the name information brand condition, and the name information generic condition (*p*s < 0.048, see Table 2 for group means).

**Table 2:** Mean (SE) warned and unwarned symptoms scores. Baseline and negative expectation ratings are raw, while model adjusted values are reported for follow-up measures.

	Name Information			Price Information		
	*Control* *(n = 30)*	*Branded* *(n = 32)*	*Generic* *(n = 30)*	*Control* *(n = 30)*	*Branded* *(n = 31)*	*Generic* *(n = 31)*
Baseline						
Warned symptoms	4.23 (0.94)	3.34 (0.77)	2.53 (0.65)	2.03 (0.56)	4.16 (0.91)	3.71 (0.84)
Unwarned symptoms	7.50 (1.22)	5.78 (0.99)	5.87 (1.03)	5.50 (0.98)	9.32 (1.40)	7.90 (1.25)
Follow-up						
Warned symptoms	2.19 (0.52)	3.01 (0.64)	4.21 (0.85)	1.20 (0.38)	1.45 (0.39)	2.60 (0.58)
Unwarned symptoms	3.87 (0.74)	3.79 (0.73)	4.44 (0.85)	2.90 (0.65)	3.73 (0.69)	2.61 (0.55)
Negative expectations	0.03 (0.03)	2.42 (0.33)	3.33 (0.41)	0.08 (0.04)	1.45 (0.24)	2.87 (0.37)

Baseline warned and unwarned symptoms scores were controlled in analyses examining their corresponding follow-up symptoms scores, as planned. Initial models including an interaction term for relevant baseline symptoms and condition were run. Baseline scores did not show a significant interaction with condition in either model, *p*s > 0.15, and thus the interaction was not retained in the final models for follow-up warned and unwarned symptoms scores.

### 2.2 Follow-up symptoms scores

#### 2.2.1 Warned symptoms

**Fig. 2:**
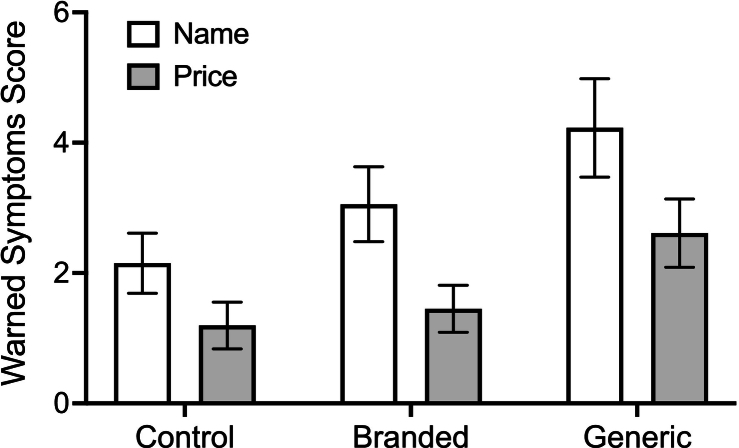
Bar graph showing follow-up mean (SE) warned symptoms scores across the three conditions, by information manipulation. Means are adjusted for baseline warned symptoms scores.

There was a statistically significant main effect of condition on follow-up warned symptoms scores, Wald *χ*^2^ = 9.39, *df* = 2, *p* = .009. The first planned (nocebo) contrast showed evidence of a significant nocebo effect, with the mean warned symptoms scores of the two sham-treated conditions (brand and generic) significantly higher than the control condition, Wald *χ*^2^ = 5.91, *df* = 1, *p* = .015, *d* = 0.28. The second planned (brand-generic) contrast showed evidence that the generic condition resulted in increased warned symptoms scores compared to the brand condition, Wald *χ*^2^ = 4.13, *df* = 1, *p* = .042, *d* = 0.36 (see Table 2 and Fig. 2). There was an unexpected significant main effect of information, Wald *χ*^2^ = 9.32, *df* = 1, *p* = .002. Collapsing across condition, those exposed to the name information manipulation reported significantly higher warned symptoms scores (*M* = 3.03, *SE* = 0.39) compared to the price information manipulation (*M* = 1.65, *SE* = 0.26; *d* = 0.43). There was not a significant information by condition interaction, *p* = .86.

#### 2.2.2 Unwarned symptoms

There was not a significant effect of condition on follow-up unwarned symptoms scores, Wald *χ*^2^ = 0.43, *df* = 2, *p* = .81, and neither planned contrast was significant, *p*s > 0.60. Similarly, there was not a significant effect of information on follow-up unwarned symptoms scores, Wald *χ*^2^ = 3.01, *df* = 1, *p* = .083, nor an interaction between condition and information, Wald *χ*^2^ = 1.82, *df* = 2, *p* = .40.

### 2.3 Negative expectations

**Fig. 3:**
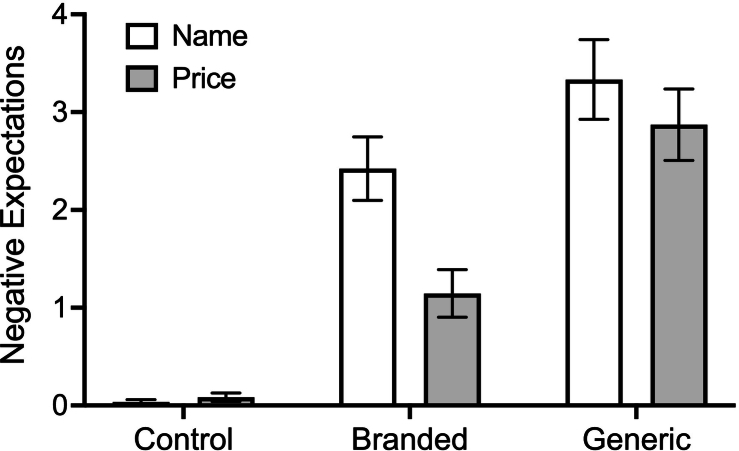
Bar graph showing mean (SE) negative expectations across the three conditions, by information manipulation.

There was a significant main effect of condition on negative expectations, Wald *χ*^2^ = 79.88, *df* = 2, *p* < .001. The first planned contrast showed that the mean negative expectations of the two sham-treated conditions (brand and generic) were significantly higher than the control condition, Wald *χ*^2^ = 201.54, *df* = 1, *p* < .001, *d* = 1.44. The second planned contrast showed that participants in the generic condition reported greater negative expectations than those in the brand condition, Wald *χ*^2^ = 12.92, *df* = 1, *p* < .001, *d* = 0.65 (see Table 2 and Fig. 3). There was not a significant main effect of information on negative expectations, Wald *χ*^2^ = 0.07, *df* = 1, *p* = .79, or an interaction between condition and information, Wald = 3.34, *df* = 2, *p* = .19.

### 2.4 Mediation of Nocebo and branded-generic effects by negative expectations

A mediation analysis was carried out to test whether the nocebo effect (i.e., higher warned symptoms scores reported by those in the sham-treated conditions compared to the no-treatment control group), and the brand-generic effect (i.e., higher warned symptoms scores reported by those in the generic compared to the brand condition), were mediated by negative expectations about side effects, controlling for baseline warned symptoms scores. The two contrasts were estimated in a single model.

**Fig. 4:**
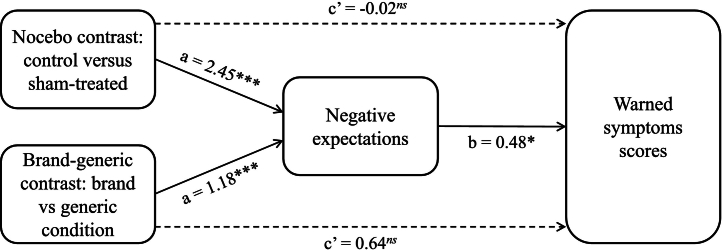
Indirect effects of nocebo and brand-generic contrasts on warned symptoms scores via negative expectations about side effects. *Note:* *p < .05; ***p < .001.

The nocebo contrast (no-treatment control versus the sham-treated conditions) showed a significant indirect effect (IE) on warned symptoms scores via negative expectations, *IE* = 1.19, *SE* = 0.63, 95 % CI = 0.02; 2.49, indicating that the observed nocebo effect was mediated by negative expectations (see Fig. 4). The brand-generic contrast (brand versus generic conditions) also showed a significant indirect effect on warned symptoms scores via negative expectations, *IE* = 0.57, *SE* = 0.32, 95 % CI = 0.01; 1.28, indicating that the higher symptoms reported in the generic compared to brand conditions were also mediated by negative expectations (see Fig. 4).

### 2.5 Branded vs generic information manipulation checks

#### 2.5.1 Perceived name complexity

Within the name information manipulation, those in the generic (complex name: “Halyazeoiipam”) sham-treated condition (*M* = 5.62, *SE* = 0.21) perceived the name of their nasal spray as significantly more complex than those in the branded (simple name: “Halpam”) sham-treated condition (*M* = 2.22, *SE* = 0.26), *t*(59) = 10.08, *p* < .001, *d* = 1.32.

#### 2.5.2 Perceived price

Within the price information manipulation, those in the generic (low price: $2.43) sham-treated condition (*M* = 1.39, *SE* = 0.16) perceived the price of their nasal spray as being significantly cheaper than those in the branded (high price: $30.33) sham-treated condition (*M* = 5.06, *SE* = 0.20), *t*(60) = 14.57, *p* < .001, *d* = 0.99.

#### 2.5.3 Perception of treatment as branded or generic

Within the name information manipulation, a large proportion of participants reported being unsure of whether the nasal spray they received was branded or generic (see Table 3). In addition, more participants across both the branded and generic conditions perceived the spray as generic than branded. There was no significant difference between the branded and generic conditions in perceptions of the spray, Pearson *χ*^2^ = 0.86, *df* = 2, *p* = .65.

**Table 3:** Number (percent) of participants who perceived the sham nasal spray as branded, generic, or unsure across the two information conditions, by branded and generic cue manipulation.

	Name Information		Price Information	
*Perception of spray*	*Branded* “Halpam” *(n = 32)*	*Generic* “Halyazeoiipam” *(n = 29)*	*Branded* “$30.33” *(n = 31)*	*Generic* “$2.43” *(n = 31)*
Branded	2 (6 %)	3 (10 %)	21 (68 %)	0 (0 %)
Generic	11 (34 %)	12 (41 %)	4 (13 %)	24 (77 %)
Unsure	19 (59 %)	14 (48 %)	6 (19 %)	7 (23 %)

Within the price information manipulation, there was a significant difference between conditions in whether the spray was perceived as branded or generic, Pearson *χ*^2^ = 35.36, *df* = 2, *p* < .001. Those in the branded condition were more likely to perceive the spray as branded, and those in the generic condition were more likely to perceive the spray as generic (see Table 3).

### 2.6 Gender and Nocebo effects

Although the literature is inconsistent,^16^^,^^41^^,^^42^ some research points to potential gender differences in nocebo effects.^43^, ^44^, ^45^ Post hoc analyses were conducted to assess the influence of participant gender on nocebo effects in warned symptoms scores. First, the eight participants who identified as nonbinary or chose not to disclose their gender were excluded, and the original model was re-run to confirm that the pattern of results did not change substantially. The pattern of results was unchanged, demonstrating a significant nocebo contrast (*p* = .026) and a significant brand-generic contrast (*p* = .031). The model was then re-run with gender included as an additional binary variable. There was a main effect of gender, Wald *χ*^2^ = 8.20, *df* = 1, *p* = .004, reflecting a well-established pattern in the symptoms literature^30^ in which female participants (*M* = 2.54, *SE* = 0.33) report significantly higher symptoms scores compared to male participants (*M* = 1.05, *SE* = 0.30). However, there were no significant interactions between gender and any other factors in the model (*p*s > 0.086), suggesting that warned symptoms scores did not differ significantly by gender across levels of information or condition in the current study.

## 3 Discussion

Results provide experimental evidence that negative expectations and subsequent nocebo effects may contribute to observed differences in treatment outcomes between branded and generic medications. The association of a sham treatment with cues typical of generic medications (low price and more complex name) resulted in an increased nocebo effect compared to when sham treatments were associated with cues typical of branded medications (high price and simple name). These results are consistent with previous findings in both active and sham treatments demonstrating that an association with generic – compared to brand – labelling can result in poorer treatment outcomes, including increased side effects.^4^^,^^5^^,^^7^^,^^8^ The presence of generic name and price cues were sufficient to increase the nocebo effect, even in the absence of explicit information that the sham treatment was a generic.

Generic treatment cues were associated with increased negative expectations about the likelihood of experiencing treatment side effects, compared to brand cues. This finding is consistent with previous research showing that both low price and complex name information are associated with heightened negative expectations.24., 25, 26 The current research extends previous work to demonstrate that these negative expectations mediated the increased nocebo effect of generic compared to brand cue-associated treatments. Cues typically associated with generic medications appear to trigger greater negative expectations which lead to enhanced nocebo effects.

In addition, the novel paradigm employed in this study successfully generated an overall nocebo effect. Similar to previous research using sham treatment paradigms to explore nocebo effects, this effect was specific to the symptoms that participants were warned about as possible treatment side effects.^12^^,^^29^^,^^43^^,^^46^^,^^47^ The results also provide further experimental support for the role of negative expectations in generating nocebo effects. While negative expectations have been theorised as a key psychological mechanism in the nocebo effect,^6^^,^^9^ relatively little experimental research has tested this causal pathway.^10^^,^^11^^,^^48^

The provision of price and name information associated with the sham nasal sprays successfully modulated the perceived cost and name complexity of the treatments. However, participants in the price groups more readily associated low price with generic, and high price with brand name, treatments. In contrast, treatments associated with a complex name were not explicitly recognised as being generic, or those with a simple name recognised as being branded. Despite this finding, there were no significant information by condition interactions, suggesting that the pattern of both negative expectations and nocebo effects did not differ by whether participants received the price or name information. Although both price and name cues appeared to shape brand vs generic differences, name cues do not appear to do so through explicit perceptions that the sham treatment was a generic. It may be that while lower price is readily recognised as being a key feature of generic medications,^49^ drug naming conventions may be less well known in the general population. Name cues may influence expectations and subsequent nocebo effects via their influence on perceived fluency,24., 25, 26 rather than via perceptions that a treatment is a brand or a generic.

The main effect of information (name vs price) on warned symptom scores was an unexpected finding. Across all conditions, those in the price condition reported significantly lower warned symptoms scores compared to those in the name condition. We propose that this effect of information may have been driven by heightened familiarity with the name ‘oxytocin’ for price information participants. The name oxytocin was used in the consent form as well as the experimenter script and subsequently when describing the sham nasal spray. In contrast, for those who received name information, the sham nasal spray was accompanied by the introduction of two novel names (Halpam and Halyazeoiipam). These relatively less familiar names may have resulted in larger nocebo symptoms due to generally heightened negative expectations and emotions across conditions, consistent with a mere exposure effect.^50^ Though notably, there was not a significant main effect of information on negative expectations. Visual inspection of Fig. 3 indicates that negative expectations differed by information (name vs price) most substantially in the braded condition. This pattern of results may reflect the additional role of negative emotions, which can also be influenced by the mere exposure effect, but were not measured in the current study.

Such familiarity effects warrant further exploration as an additional contributing factor to increased nocebo effects associated with generic medications. In medical consultations, physicians use generic drug names significantly less frequently than brand names when discussing medications with patients.^51^ Communications within healthcare settings also follows this pattern, with written notes referencing brand rather than generic medication names at a ratio of 100 to 1.^52^ Patient familiarity with brand names may also be influenced by media reporting. News coverage referencing medications is also likely to exclusively or preferentially use brand names compared to generic names.^53^ Lack of exposure to and familiarity with generic names in clinical encounters or via media consumption may lead to increased nocebo effects. Increasing exposure to generic names could offer a pathway to reducing nocebo effects.

The current study is strengthened by the inclusion of a no treatment control group, explicit assessment of negative expectations, and experimenter blinding. There are also some limitations to note. The price and name cues were manipulated independently, limiting the ecological validity of the design. However, the consistent pattern of results across information conditions supports the contribution of both of these types of cues as contributors to nocebo effects associated with generic medications. Due to ethical issues around inducing nocebo effects in patient populations, healthy general population participants were recruited, and the treatment used was not medically necessary or likely to induce lasting improvements in health or quality of life. However, nocebo effects occur in both healthy participants^54^ and in a range of clinical contexts,^55^ and evidence suggests that the mechanisms underlying the nocebo effects are consistent across both healthy^56^ and patient populations.^57^ The sample was also predominantly young and well educated, thus it is unclear how the current findings might generalise to more diverse populations. However, some evidence indicates that nocebo effects may be larger in older and less educated populations,^58^ meaning that the current results could underestimate the impact of generic medication cues on nocebo effects in the wider population. Although no significant gender differences were found in the nocebo effect in the current study, the group sizes for these analyses were small, and the study was not designed or powered to detect this effect. The primary nocebo outcome was self-reported symptom experience, and may thus be subject to reporting bias. However, self-reported symptoms drive perceptions of health and treatment-related behaviours^59^^,^^60^ and thus warrant empirical consideration.

Future research is needed to further explore the aspects of the treatment context that contribute to increased nocebo effects associated with generic medications. Treatment familiarity, other treatment cues including medication packaging as well as the potentially additive nature of multiple cues, and how physicians and pharmacists communicate with patients about branded and generic treatments all warrant investigation. Further development and testing of interventions to improve patient expectations of generics, and subsequent treatment outcomes, is also needed. Evidence on the benefits of patient education is mixed, with some research showing positive effects from education about the equivalence and approval process for generics,^61^ while other research shows detrimental effects of such interventions on treatment outcomes.^62^ Outside of educational interventions, positive framing of side effect information may be useful to mitigate nocebo effects from generic treatments.^63^

Generic medications are central to providing both individual patients and healthcare systems with affordable access to a wide range of medications. Generics are held to strict quality standards and are required to be equivalent in both safety and efficacy to their brand name counterparts, and under double blind conditions this is usually the case. However, when patients know they are taking a generic, this can result in reduced treatment benefits and increased side effects. The current study provides experimental evidence indicating that increased side effects from generic compared to brand name drugs can be caused by the nocebo effect. Cues typically associated with generics can trigger negative expectations and subsequent nocebo side effects. These findings have relevance both to medical care and public health policy, and future research on reducing nocebo effects from generic medications is needed.

## CRediT authorship contribution statement

**Kiarne Humphreys:** Writing – review & editing, Writing – original draft, Project administration, Methodology, Investigation, Formal analysis, Data curation, Conceptualization. **Michelle Lin:** Writing – original draft, Project administration, Methodology, Investigation, Formal analysis, Data curation, Conceptualization. **Kirsten Barnes:** Writing – review & editing, Supervision, Project administration, Formal analysis, Data curation. **Yasmin Hasan:** Writing – review & editing, Investigation. **Ashwin Vignaraja:** Writing – review & editing, Investigation. **Kritika Sarna:** Writing – review & editing, Investigation. **Andrew L. Geers:** Writing – review & editing, Resources, Methodology, Funding acquisition, Conceptualization. **Kate Faasse:** Writing – review & editing, Writing – original draft, Visualization, Supervision, Resources, Project administration, Methodology, Funding acquisition, Formal analysis, Data curation, Conceptualization.

## Funding

This work was supported by the 10.13039/501100000923Australian Research Council [DP220102231].

## Declaration of competing interest

The authors declare that they have no known competing financial interests or personal relationships that could have appeared to influence the work reported in this paper.

## Appendix A Supplementary data

Supplementary material Image 1
