# Supplementary material for: Not so great expectations: The role of price and name information in the nocebo effect
Source: Explor Res Clin Soc Pharm. 2025 Jun 28;19:100630. doi: 10.1016/j.rcsop.2025.100630 (PMC12274324; doi:10.1016/j.rcsop.2025.100630)
Supplement: Supplementary file 1 — Supplementary material [file mmc1.docx]

**Online supplementary materials: the role of price and name information in the nocebo effect**

**Appendix 1: Pilot Study**

A pilot study was conducted to investigate which names and price points were appropriate for the main study. Participants (*N* = 100) were recruited through the Prolific Academic platform to complete an online questionnaire and received £1 as recompense for 10 minutes of their time. After completing demographic questions, participants were instructed to imagine that a pharmaceutical company had developed a nasal spray containing oxytocin that improved feelings of trust and cooperation. Participants were provided with this information as it aligns with the cover story of the main study.

**Price Questions**

Participants were asked to respond to two hypothetical scenario questions. The hypothetical scenarios described two oxytocin nasal sprays: one low-quality nasal spray and one high-quality nasal spray. The low-quality spray scenario described that the nasal spray was made using low-quality ingredients where the manufacturers cut corners. The high-quality spray scenario described that a strict manufacturing process was followed, and the nasal spray was produced using the highest quality ingredients. Participants were asked to rate how much they would expect to pay for each nasal spray using a slider that ranged from $0 to $100 (see Table 1 for means and standard deviations).
**Table 1.** *Mean price response for pilot study scenario questions*

|  | Low-price scenario | High-price scenario |
| --- | --- | --- |
| *Mean* | $8.08 | $19.13 |
| *Standard deviation* | $5.65 | $11.20 |
| *Final price* | $2.43 | $30.33 |

To ensure that the two prices used for the main study were distinct from one another, one standard deviation was subtracted from the low-price value and one standard deviation was added to the high-price value (see Table 1). Extending the range between the two prices was done to try to ensure participants in the main study perceived the low-price treatment as 'cheap’, and the high-price treatment as 'expensive'.

**Name Questions**

Perceptions of four fictitious oxytocin medications with different names (Temolin, Theophylliane, Halpam, and Halyazeiiopam) were assessed. These medication names were chosen from Cho's (2015) study, as they were designed using linguistic algorithms to depict different levels of "processing fluency" and have previously created different expectations in fictitious medication (Oppenheimer, 2006; Travers & Oliver, 1978). Following this information, participants answered 10 questions about each medication's name. For each name, participants were asked to read and pronounce the name of the medication out loud before answering each question on a 6-point Likert scale. Three of these questions were of primary interest for this study; they asked about name complexity, length, and ease of pronunciation, as these factors constitute a complex "generic" medication name and aligns with processing fluency theory (Faasse & Martin, 2018; Oppenheimer, 2006). The seven remaining questions were included to gain additional information about how names were perceived; they asked about name familiarity, liking, perceived effectiveness, perceived risk, the likelihood of side effects, willingness to use, and perceptions of branding (Dohle & Siegrist, 2014; Song & Schwarz, 2009). The four medication names were presented in random order to counteract order effects.

An *overall complexity score* was calculated for each medication name using SPSS

version 26. This overall complexity score reflected the mean of complexity, length, and ease-of-pronunciation ratings, where higher scores indicated that the name was perceived as more complex. Overall complexity scores were compared for the four names; the results indicated that Halpam was perceived as the least complex, and Halyazeiiopam was perceived as the most complex (see Figure 1). Thus, these two names were selected for the branded and generic conditions, respectively.

**Figure 1.** *Bar graph showing mean (SD) overall complexity scores as a function of medication* *name*


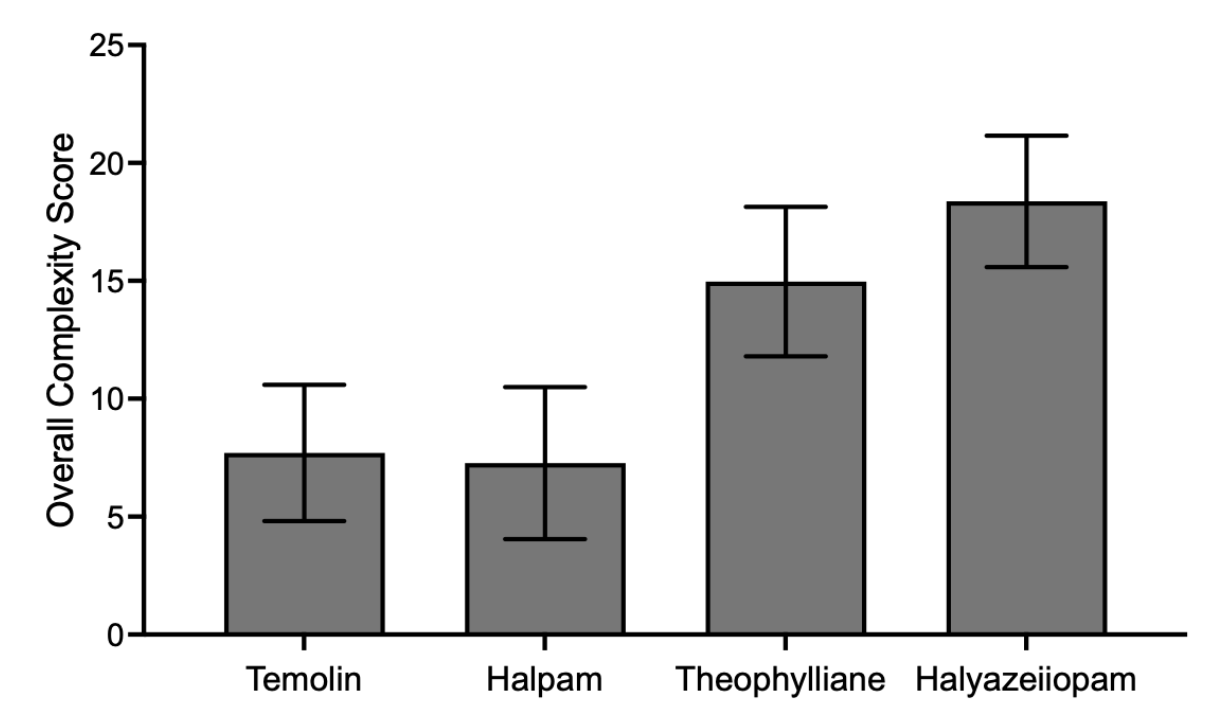


**References**

Cho, H. (2015). The malleable effect of name fluency on pharmaceutical drug perception. *Journal of health psychology, 20*(10), 1369–1374. <https://doi.org/10.1177/1359105314525486>

Dohle, S., & Siegrist, M. (2014). Fluency of pharmaceutical drug names predicts perceived hazardousness, assumed side effects and willingness to buy. *Journal of Health* *Psychology, 19*(10), 1241–1249. <https://doi.org/10.1177/1359105313488974>

Faasse, K., & Martin, L. R. (2018). The power of labeling in nocebo effects. *International* *Review of Neurobiology, 139*, 379–406. <https://doi.org/10.1016/bs.irn.2018.07.016>

Oppenheimer, D. M. (2006). Consequences of erudite vernacular utilized irrespective of necessity: Problems with using long words needlessly. *Applied Cognitive Psychology,* *20*(2), 139–156. <https://doi.org/10.1002/acp.1178>

Song, H., & Schwarz, N. (2009). If it’s difficult to pronounce, it must be risky: fluency, familiarity, and risk perception. *Psychological Science, 20*(2), 135–138. <https://doi.org/10.1111/j.1467-9280.2009.02267.x>

Travers, J., & Olivier, D (1978). Pronounceability and statistical ‘Englishness’ as determinants of letter identification. *American Journal of Psychology, 91*(3), 523-538. <https://doi.org/10.2307/1421698>
